# SE-Attention Augmented Hybrid CNN–BiLSTM Model for Leakage Current-Based Detection of Cracked and Broken High-Voltage Porcelain Insulators

**DOI:** 10.3390/biomimetics11070457

**Published:** 2026-07-01

**Authors:** Ömer Faruk Alçin, Muhammed Buğracan Özküçük, Muhsin Tunay Gençoğlu

**Affiliations:** 1 Department of Software Engineering, Faculty of Engineering, Inonu University, 44210 Malatya, Türkiye; 2 Department of Electrical and Electronics Engineering, Faculty of Engineering and Natural Sciences, Malatya Turgut Ozal University, 44210 Malatya, Türkiye; bugracan.ozkucuk@ozal.edu.tr; 3 Department of Electrical and Electronics Engineering, Faculty of Engineering, Firat University, 23119 Elazığ, Türkiye; mtgencoglu@firat.edu.tr

**Keywords:** porcelain insulator fault diagnosis, leakage current signals, CNN–BiLSTM, Squeeze-and-Excitation attention, deep learning, feature extraction

## Abstract

Extreme and sudden temperature fluctuations observed as a result of global climate change increase the environmental pressure on energy transmission infrastructure. These meteorological changes significantly increase the risk of failure for porcelain insulators, which exhibit low thermal resistance and are susceptible to sudden arcing and surface deformations. In this study, a hybrid CNN–BiLSTM–SE architecture augmented with the Squeeze-and-Excitation attention mechanism is proposed using surface leakage current signals to diagnose healthy, cracked, and broken structural conditions in three-unit porcelain insulators. The SE block in the architecture dynamically rescales feature maps from CNN layers on a channel-by-channel basis. Thus, it highlights the signal characteristic that is dominant for fault diagnosis just before the BiLSTM units learn temporal dependencies. Leakage current data were obtained under an experimental setup at 60 kV for 15 different conditions covering all possible combinations of healthy, cracked, and broken insulator units. The raw signals were preprocessed with the Savitzky–Golay filter to suppress noise while preserving the diagnostic waveform morphology. 24 features covering time-domain statistics, frequency-domain spectral characteristics, and wavelet-domain energy components were extracted and used as model inputs. The CNN–BiLSTM–SE architecture achieved a classification accuracy of 93.83%, surpassing the standalone CNN (88.89%), BiLSTM (87.65%), and CNN–BiLSTM (91.36%) models, as well as classical machine-learning baselines (SVM: 87.65%, Random Forest: 90.12%, Boosted Trees: 87.65%).

## 1. Introduction

The reliability of high-voltage transmission infrastructure is increasingly challenged by climate change: extreme and rapid temperature fluctuations impose growing thermal stress on outdoor insulators, and recent studies report a rising incidence of weather- and temperature-related insulation failures [1,2]. The stable operation of the production, transmission, and distribution phases of electrical energy is critically important for today’s societal structure and industrial continuity. High-performance insulators enhance the overall safety and stability of the power system. However, insulators can carry a serious risk of failure when exposed to outdoor conditions for extended periods. Porcelain insulators have operational limitations due to their low thermal resistance, high maintenance costs, and the difficulty in detecting structural defects. Furthermore, failure types such as electrical breakdown, flashover, and surface cracking/breaking are frequently observed in these insulators [3]. Studies in the literature show that more than 75% of failures occurring in electrical grids are caused by insulator defects [4,5,6]. Therefore, periodic inspection of insulators is a critical requirement for maintaining the stability and operational reliability of power systems.

Methods available for detecting insulator defects include observation, ultrasonic detection, ultraviolet pulse, electric field, conventional image processing, infrared imaging, and the leakage current measurement method used in this study. Visual inspection methods [7], while inexpensive and easy to use, have disadvantages such as low efficiency and a high error rate. The ultrasonic detection method [8], although fast and low-cost, only detects mechanical failures. The ultraviolet pulse method [9], although highly sensitive, consists of expensive equipment that is sensitive to temperature changes. The electric field method [10], although precise in measurement, is used only for single-type detection (detecting defective insulators). Although traditional image processing techniques [11] offer low implementation costs and operational simplicity, they exhibit limited detection accuracy under complex backgrounds and variable environmental conditions. The infrared imaging method [12], although simple to use and providing clear results, is expensive and sensitive to temperature. The leakage current measurement method is used to detect various types of faults on the insulator surface that cause insulator malfunctions, such as contamination, icing, breakage, and cracking [13,14,15]. Negative factors such as contamination on the insulator surface, moisture, and structural deformation cause an increase in leakage current amplitude. Therefore, monitoring the leakage current magnitude serves as a reliable indicator for determining the dielectric condition and degradation level of the insulator. This method has significant advantages in that it is resistant to environmental variables and offers real-time status monitoring capabilities [16].

In the study where data was collected using acoustic sensors by applying 40 kV voltage to a faulty porcelain insulator, artificial neural networks were used [17]. A 96.03% accuracy rate was achieved using wavelet transform analysis, whereas an 88.65% accuracy rate was attained with fast Fourier transform analysis. A single-shot multi-box detector (SSD) was employed to identify porcelain and silicone insulators in aerial pictures [18]. Background and type differences affect detection accuracy in insulator identification; average sensitivity values for porcelain and silicone insulators were 94.12% and 86.70%, respectively, in forest backgrounds and 90.51% and 87.29% in complex backgrounds containing buildings. A method based on ResNeSt and Region Proposal Network (RPN) has been proposed for the detection of insulator defects [19]. The model achieves a 98.38% accuracy rate in insulator fault detection and can process 12.8 images per second. A VGG-19-based transfer learning method has been proposed to improve the detection of insulator defects in high-voltage transmission lines [20]. The pre-trained VGG-19 model was trained using the ImageNet dataset, and this knowledge was transferred and applied to determine whether insulators were missing or damaged. A method combining Gray Level Co-occurrence Matrix (GLCM) with RGB color space statistical prior information was proposed to filter out complex backgrounds and varying light conditions [21]. The method’s mAP showed an improvement of approximately 4.1 percentage points compared to the best-performing baseline method.

In recent years, there has been a significant increase in the trend towards the use of Convolutional Neural Networks (CNN) and YOLO (You Only Look Once)-based deep learning architectures for insulator fault diagnosis and classification in the literature [22,23]. A method for detecting insulators with minor faults in transmission line images featuring intricate backgrounds is proposed, utilizing an enhanced YOLOv7 algorithm [24]. The testing results indicate that the model attained an accuracy of 93.8%, surpassing the performance of Faster R-CNN, YOLOv7, and YOLOv5s by 7.6%, 3.7%, and 4%, respectively. The YOLOv8 algorithm is presented for the precise identification of insulators and defective regions in aerial photos with intricate backgrounds [25]. A model utilizing GhostNet and multi-scale asymmetric convolution (MSA-GhostBlock) was employed to enhance target feature extraction in intricate background images. The overall accuracy rate of the proposed model was determined to be 93.9%. A new method called Faster R-Transformer, combining the advantages of Convolutional Neural Networks (CNN) and the self-attention mechanism of Transformers, has been proposed for the effective detection of insulators in complex aerial environments [4]. The proposed faster R-transformer architecture achieved a 97.31% accuracy rate in insulator detection. A convolutional neural network (CNN) using small samples, data augmentation, and transfer learning has been proposed for insulator fault detection [26]. Within the scope of the study, a comprehensive dataset consisting of 3000 samples was obtained by enriching a limited number of 200 insulator images with data augmentation methods. As a result of training with a VGG16-based transfer learning network, a high accuracy value of 98.71% was achieved.

Existing algorithms for detecting insulator defects face significant limitations due to high computational costs and time requirements in processing large-scale raw images. The limited and heterogeneous nature of publicly available datasets in the literature makes it difficult to train and generalize these models comprehensively [3,5]. While the representation of objects with a limited perspective, particularly in aerial images, leads to the loss of critical attributes, processing high-resolution data requires significant computing power [27]. Additionally, object overlaps encountered in image-based methods, complex background noise, and subjective errors in data labeling processes negatively affect diagnostic performance. These image processing-based challenges and operational obstacles in dataset creation have necessitated the use of alternative data sources that are more reliable and based on direct physical measurements. In this context, the use of leakage current data that directly reflects the electrical condition of the insulator not only offers a diagnostic mechanism less affected by environmental variables but also provides more reliable condition monitoring by eliminating the high processing load and complexity created by visual data sets.

This study proposes an explainable hybrid deep learning framework that integrates Convolutional Neural Networks (CNN), Bidirectional Long-Short-Term Memory (BiLSTM), and a Squeeze-and-Excitation (SE) channel attentional mechanism for the accurate detection of cracks and fracture structural distortions in porcelain insulators. The proposed framework draws inspiration from biological information processing. The SE attention mechanism is conceptually inspired by the selective attention of biological neural systems: just as the human visual and nervous systems allocate processing resources preferentially to the most salient stimuli, the SE block adaptively emphasizes the most informative feature channels and suppresses less relevant ones [28]. This is combined with a bidirectional recurrent structure (BiLSTM) that emulates the way biological memory integrates information from both past and future context. Through these biologically inspired mechanisms, the study applies a bio-inspired learning paradigm to a real-world environmental degradation problem. In experimental studies, characteristic leakage current data under high voltage were obtained for scenarios representing the healthy, cracked, and broken structural conditions of three-unit porcelain insulators. The obtained leakage current signals were pre-processed with the Savitzky–Golay filter to remove noise and preserve waveform continuity; subsequently, a total of 24 distinctive features were extracted from the time domain, frequency domain, and wavelet domain. The extracted features were applied as inputs to CNN, BiLSTM, and the proposed CNN–BiLSTM–SE architectures for performance comparison purposes. Experimental results reveal that the proposed hybrid architecture offers superior accuracy in insulator fault diagnosis compared to other models. The key contributions that constitute the original value of this article are summarized below:

(1) A bio-inspired hybrid architecture is proposed that integrates a Squeeze-and-Excitation channel-attention mechanism into a CNN–BiLSTM backbone for leakage-current-based insulator diagnosis. Unlike existing CNN–LSTM/BiLSTM leakage-current models, the SE block adaptively recalibrates the feature channels, and an ablation study quantifies the contribution of each component.
(2) The model’s decisions are validated and interpreted through a combined statistical explainability framework. The Kruskal–Wallis test and Fisher Discriminant Ratio establish the discriminative power of the extracted features, while permutation- and occlusion-based attribution reveal that the model relies on physically meaningful signal characteristics.
(3) A characteristic dataset consisting of 15 different scenarios encompassing all possible combinations for healthy, cracked, and broken states was designed for a three-unit porcelain insulator, with cracks induced by a thermal-shock procedure that emulates climate-driven thermal stress.

## 2. Materials and Methods

### 2.1. Experimental Setup

**Figure 1:**
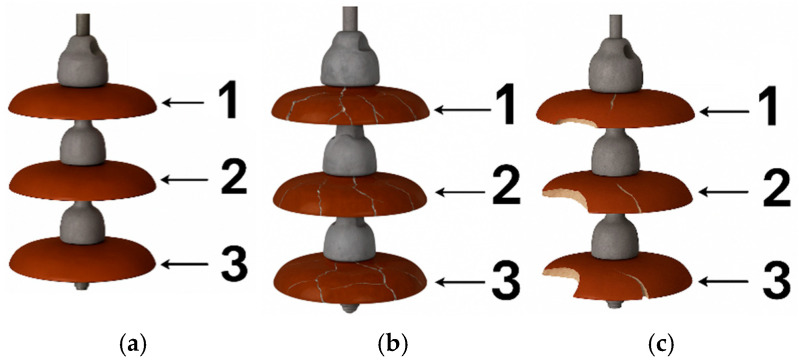
Porcelain insulators with different morphological structures: (**a**) healthy; (**b**) cracked; (**c**) broken.

In porcelain insulators, low temperature resistance leads to extensive surface cracking. Over time, crack formation progresses, weakening structural integrity and causing breakage. Therefore, it is important to examine cracked and broken insulators together. Healthy porcelain is defined as an insulator that does not have any form of defect (such as breakage, cracking, contamination, or icing) on its surface. The three different insulator conditions examined are presented in Figure 1.

The defective units were prepared under a controlled laboratory procedure. Cracked units were produced by a thermal-shock process in which the cooled insulator surface was rapidly immersed in hot water, followed by light mechanical taps to the initial. Superficial surface cracks without any loss of material. Broken units were produced by direct mechanical impact, resulting in the physical detachment of a porcelain fragment from the insulator body.

In the experimental studies, U100 BL manufactured in Ankara, Türkiye, and type-tested in accordance with TS EN 60383-1 at a high-voltage test laboratory accredited by the Turkish Accreditation Agency (TÜRKAK) porcelain insulators were used. The basic geometric properties of the insulator are as follows: disc diameter (D) 255 mm, height (H) 146 mm, and pin head diameter (d) 16 mm.

Fifteen such situations were defined in the experimental experiments to encompass all potential fault combinations that may arise in a three-unit porcelain insulator array. The situations are detailed in Table 1.

**Figure 2:**
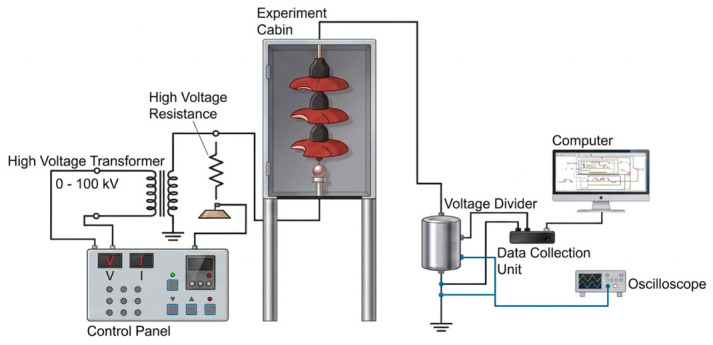
Experimental high-voltage setup for the acquisition of insulator leakage current signals.

In the literature, a voltage of 40 kV was applied to a single-unit porcelain insulator [17]. Structural deformations on the insulator surface directly affect the amplitude and harmonic content of surface leakage currents (LC). In this study, a high voltage of 60 kV was applied to the insulator array to make the leakage current characteristics more distinct and to increase diagnostic sensitivity. The laboratory-scale test setup prepared for obtaining leakage current data is schematized in Figure 2.

The high-voltage transformer in the test setup operates in the 0–100 kV range. Leakage current signals were systematically recorded via the National Instruments NI USB-6009 Data Acquisition (DAQ) module, which features a 14-bit resolution analog input and a USB-based interface, with a sampling interval of 1.5 ms (667 Hz sampling frequency) using the LabVIEW platform (version 2021). This sampling frequency adequately resolves the low-order harmonic distortion (up to the 5th harmonic, 250 Hz) of the 50 Hz fundamental, which carries the diagnostic fault signature, in accordance with the Nyquist criterion (Nyquist frequency = 333.3 Hz). The use of low-order odd harmonics, particularly the 3rd and 5th, as reliable indicators of insulator surface condition is well established [13,29,30].

Since the classification performance of deep learning models directly depends on the dataset size [31,32], each case was recorded as a continuous time-series of 100,000 samples. This scale of dataset enabled high generalization capacity to be achieved without the need for synthetic data augmentation. These 100,000 values per case correspond to consecutive samples of a single continuous, non-stationary acquisition that captures the time-varying discharge activity, rather than repeated identical measurements. The continuous records were subsequently segmented into model-input instances, and the dataset partition was performed at the block level prior to segmentation to prevent data leakage. The selected data volume was chosen to balance model training accuracy and computational cost.

### 2.2. Dataset Validation

**Figure 3:**
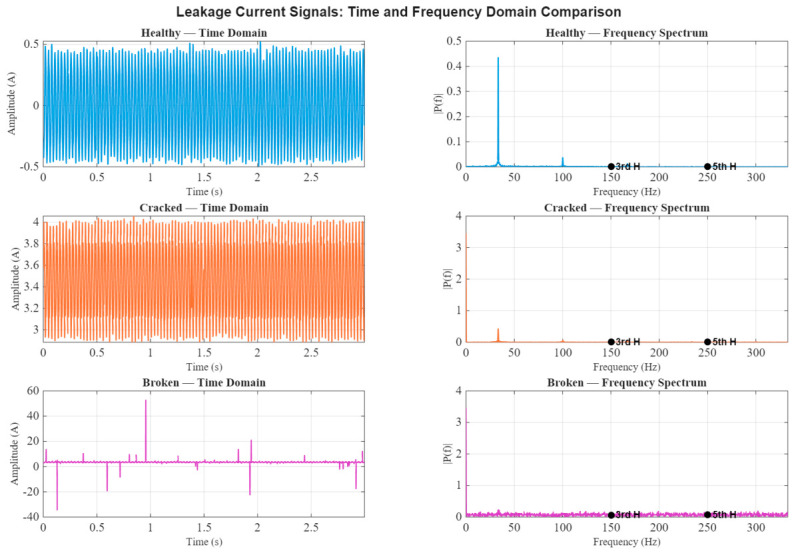
Time-domain waveforms of leakage current signals and corresponding frequency spectra for healthy, cracked, and broken porcelain insulators.

Breaks and cracks in porcelain insulators interfere with the distribution of the electric field, modifying the properties of leakage current. Figure 3 presents a comparative analysis of the time-domain waveforms and frequency spectra of the leakage current signals obtained for three different insulator cases.

When the time domain is examined, it is observed that the healthy insulator exhibits a smooth and periodic waveform within a narrow amplitude range. In the cracked insulator, the periodic structure is preserved, but the amplitude is observed to increase significantly, and a slight asymmetry appears in the waveform. In the case of a broken insulator, the waveform becomes completely irregular, and spikes caused by sudden and severe discharge appear. When examining the frequency spectrum, it is observed that in the healthy insulator, the energy is largely concentrated at the fundamental frequency (50 Hz), and harmonic components remain negligible. In the cracked insulator, it is noteworthy that the 3rd harmonic component significantly increases. In the case of a broken insulator, the energy is dispersed over a wide frequency band, and the harmonic components become indistinct. Micro discharges and arc formations on the surface of the porcelain insulator cause characteristic distortions in the waveform of the leakage current and increase the amplitudes of harmonic components. This situation directly illustrates that amplitude-based analysis alone will be insufficient and that the harmonic content plays a decisive role in fault type detection.

The validity of experimental research performed on 15 distinct data sets must be established using sophisticated statistical analyses, including data distribution assessment, variance homogeneity evaluation, and significance testing of intergroup differences. The statistical characterization of the data set within the scope of the Lilliefors normality and Levene’s homogeneity of variance tests was applied primarily. For amplitude and dominant harmonic components (3rd and 5th), a non-parametric Kruskal–Wallis analysis was performed to determine the group differences on the basis; the level of the differences obtained was quantified by effect size analysis.

To determine the statistical significance of leakage current data in fault diagnosis, differences between conditions were tested based on the null hypothesis [33]. In this analysis, the *p*-value was used as a criterion to establish whether the observed signal characteristics were random or not. The *p* < 0.05 values obtained from the Lilliefors and Levene tests indicate that the data do not exhibit normal distribution and that the group variances are not homogeneous [34,35]. However, in the Kruskal–Wallis test, a *p*-value < 0.05 indicates a statistically significant difference between at least one of the groups [36]. Micro discharges and arc formations on the surface of porcelain insulators cause characteristic distortions in the waveform of the leakage current [29,37]. Since this condition triggers a significant increase in the amplitudes of harmonic components in the frequency spectrum, restricting the Kruskal–Wallis analysis solely to the fundamental current amplitude may limit the distinguishability of faults. Therefore, it is important to include harmonics in the statistical analysis. While the *p*-value in statistical analysis assesses whether the observed difference is statistically significant, it does not reflect its practical or physical significance, namely the effect size. To overcome this limitation, Effect Size analysis is applied to determine which electrical parameters are most dominant and discriminatory in damage diagnosis [38]. The obtained η^2^ coefficients were evaluated in three main categories: small for 0.01 ≤ η^2^ < 0.06, medium for 0.06 ≤ η^2^ < 0.14, and large for η^2^ ≥ 0.14.

### 2.3. Training Process and Evaluation Metrics

The experimental studies were performed on a workstation equipped with a 14700HX CPU (Intel, Santa Clara, CA, USA), 32 GB of RAM, and an RTX PRO 2000 Blackwell GPU (NVIDIA, Santa Clara, CA, USA) with 8 GB of VRAM. The software environment was developed using MATLAB R2025a for signal processing and deep learning model implementation.

The success level of the model developed to classify crack and breakage conditions on porcelain insulator surfaces is evaluated by comparing the model predictions with actual labels. The diagnostic capability of the proposed model is based on its performance in differentiating between healthy and defective (cracked/broken) insulator classes. This situation is defined through four basic parameters [39,40]. True positive (*A*) instances are those where the model says “defective” and the insulator is indeed defective. This is the case where the system successfully detects the defect. True negative (*B*) models are examples where the model says “healthy” and the insulator is indeed healthy. This is the case where the system correctly identifies the healthy component. False positive (*C*) models are examples where the model says the insulator is “defective” but the insulator is actually healthy. False negative (*D*) models are examples where the model says the insulator is “healthy,” but the insulator is actually defective. The performance of the proposed architecture was evaluated based on five different performance metrics: accuracy, sensitivity, specificity, precision, and F1-score. The mathematical expressions for these evaluation metrics are presented in Equations (1)–(5).

(1) $$Accuracy=\frac{A+B}{A+B+C+D}$$

(2) $$Sensitivity=\frac{A}{A+D}$$

(3) $$Specificity=\frac{B}{B+C}$$

(4) $$Precision=\frac{A}{A+C}$$

(5) $$F1\mathrm{-s}core=\frac{2\times Sensitivity\times Precision}{Sensitivity+Precision}$$

## 3. Proposed Hybrid Deep Learning Architecture for Cracked/Broken Porcelain Insulator Detection

### 3.1. Signal Pre-Processing

In the diagnosis of insulator defects, leakage current measurement is of critical importance for real-time monitoring of the condition. The leakage current waveform contains both the power-frequency fundamental and its harmonic components, together with transient fluctuations that characterize the discharge development. Low-pass filters obtain leakage current pulses that directly indicate the insulator’s fault condition by taking the difference between the actual raw signal and the filtered fundamental waveform [41].

The Savitzky–Golay (SG) filter is a filter based on the principle of fitting a local polynomial with the least-squares method to data points located in a moving window. The mathematical expression of the method is as stated in Equation (6) [42].

(6) $${\hat{y}}_{m}=\sum_{k=0}^{p}a_{k}m^{k}$$

where ${\hat{y}}_{m}$ is the estimated signal value at point m, p is the degree of the polynomial, $a_{k}$ are the polynomial coefficients calculated by the least squares method, and $m^{k}$ is the kth power of point m.

Unlike standard low-pass filters, the SG filter prevents the blurring of sharp transitions in the signal by incorporating high-frequency components into the polynomial fitting process instead of directly eliminating them [43]. The flexibility in adjusting window length and polynomial degree allows the filter to adapt to diverse frequency structures, establishing a superior bias-variance tradeoff that effectively suppresses noise while capturing rapid temporal variations [44]. Since the SG filter performs flattening of the distinctive details and peak values in the signal without deforming, it increases the sensitivity of fault diagnosis by offering a much higher signal-to-noise ratio (SNR) compared to conventional methods [42]. In this study, the SG filter was applied with a polynomial order of 7 and a frame length of 21 samples. This configuration suppresses high-frequency measurement noise while preserving the discharge-pulse morphology and the low-order harmonic content—the 50 Hz fundamental and its 3rd (150 Hz) and 5th (250 Hz) harmonics—which lie well within the filter’s passband. The boundary samples are handled by polynomial fitting over the terminal frames, avoiding edge distortion.

**Figure 4:**
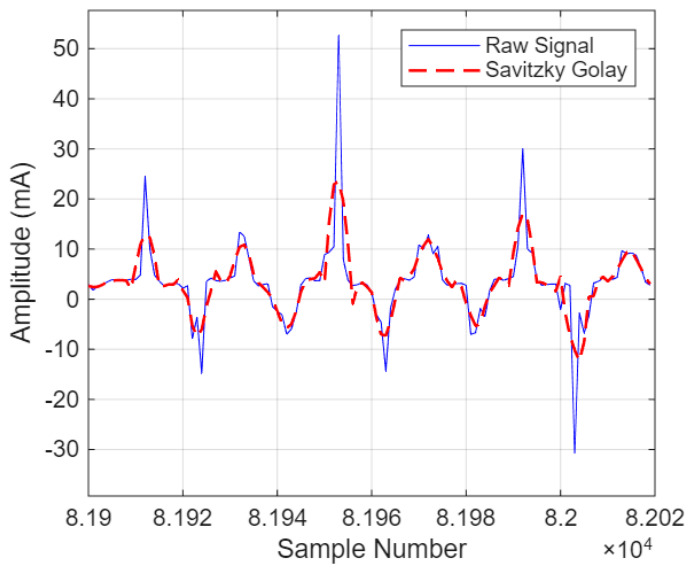
Comparison of raw leakage current data and the signal form after Savitzky–Golay filtering.

Figure 4 shows the SG filter applied to the leakage current pulses characterizing the discharge development on the surface of a defective porcelain insulator.

When examining the raw signal (blue solid line), high-frequency oscillations and sharp transitions superimposed on the overall signal shape are observed. After processing with the SG filter (red dashed line), it is observed that unwanted noise components in the signal are effectively suppressed. The noise reduction process performed increased the distinguishability of transient components characterizing discharge events within the signal.

### 3.2. Feature Extraction

When leakage currents increase on the surface of the porcelain insulator, it leads to certain changes in the amplitude, phase, and spectral content of the current waveform. The feature extraction process optimizes the decision-making mechanism of deep learning models by this dimensional reduction and feature selection, reducing the training complexity of the model while increasing its classification accuracy and generalization capability.

**Figure 5:**
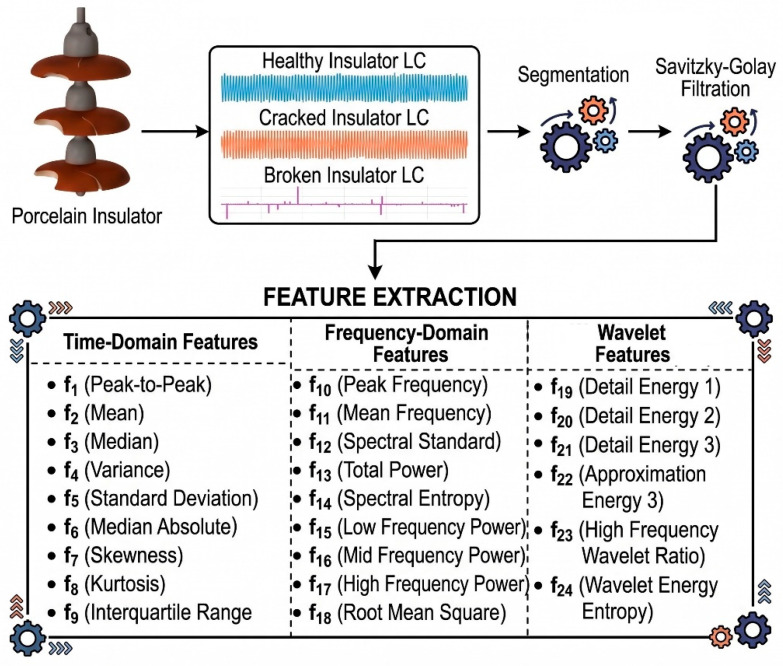
Representation of time-domain, frequency-domain, and wavelet features extracted from leakage current signals of porcelain insulators.

In the literature, the success of leakage current monitoring systems is based on the capacity of the features used to define fault characteristics [45,46]. The set of 24 features identified in this study is presented in Figure 5.

**Figure 6:**
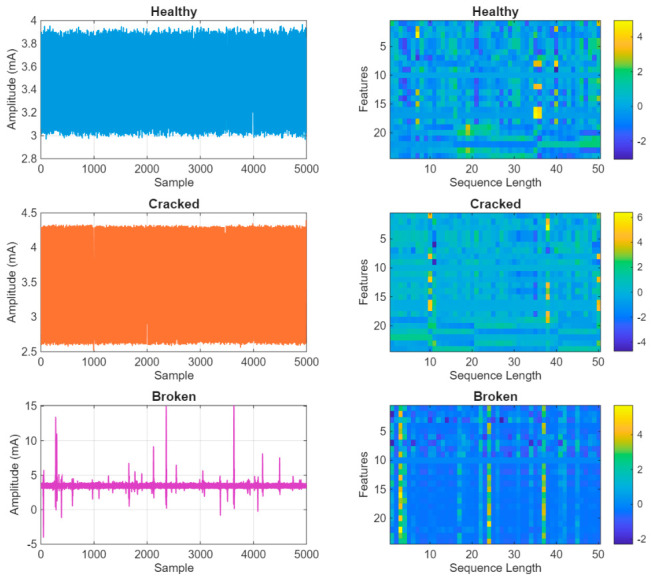
Leakage current signal segments and corresponding normalized feature matrices for healthy, cracked, and broken porcelain insulator conditions.

After preprocessing the raw leakage current signals with the Savitzky–Golay filter, the feature extraction stage begins. At this stage, the signal was first divided into 5000-point windows with a step size of 2500 points; then each window was further divided into 50 equal sub-windows, and 24 features were calculated from each sub-window. This windowing produced a total of approximately 819 feature-matrix instances across all configurations. These instances were then divided into the training, validation, and test subsets, with the 10% test partition corresponding to approximately 27 instances per class. Figure 6 presents the leakage current signals obtained for three different insulator states and the corresponding normalized feature matrices.

It is observed that in the healthy insulator, the attribute matrix exhibits a relatively homogeneous distribution; in the cracked insulator, concentration begins in certain attribute rows; and in the broken insulator, the attribute matrix assumes a distinctly heterogeneous and irregular structure. The obtained feature matrices were subjected to z-score normalization to prevent overfitting and to make features of different scales comparable.

Time-domain attributes directly characterize the temporal amplitude variations and central tendency statistics of leakage current signals. The sudden spikes and transient fluctuations resulting from discharges on the insulator surface can be captured with high precision through time-domain characteristics. The attributes used in this context are detailed in Table 2 along with their mathematical definitions and functional roles in defect detection [47,48].

Frequency domain attributes are of critical importance in terms of analyzing the harmonic distortions of leakage currents on the basic network frequency and spectral energy distribution. The interaction of capacitive and inductive components in leakage current paths significantly alters the frequency content of the signal in a manner specific to the fault type. The frequency plane attributes used in this study, along with their mathematical formulations, technical definitions, and characteristic roles in fault diagnosis, are presented in Table 3 [47,49].

Wavelet domain features play a complementary role when Fast Fourier Transformation alone proves insufficient due to the transient discharge components in leakage current signals being localized both in time and frequency [50,51]. The wavelet transform captures both high-frequency transient events and low-frequency structural changes simultaneously by decomposing the signal into detail coefficients (D1, D2, D3) and an approximation coefficient (A3) at three decomposition levels. The wavelet features used in this study are presented in Table 4.

### 3.3. SE Attention-Augmented Hybrid CNN–BiLSTM Architecture

Leakage current data exhibits both nonstationary and highly nonlinear characteristics due to the effects of physical damage [42,52]. CNN layers have the ability to learn non-linear spikes and discharge characteristics in the signal in local patterns without the need for complex mathematical calculations. On the other hand, BiLSTM units process the signal in both forward and backward directions, evaluating temporal dependencies bidirectionally. This approach provides the model with not only the instantaneous amplitude of a discharge impulse in nonstationary data but also contextual information about which environmental cycle that discharge develops in [53,54]. However, in standard CNN + BiLSTM architectures, all channels are processed with equal weight; this leads to channels that are critically important for error diagnosis not being adequately emphasized. To overcome this limitation, the proposed architecture was developed by integrating the Squeeze-and-Excitation (SE) channel attention mechanism between CNN-based spatial feature extraction and BiLSTM-based temporal modeling.

**Figure 7:**
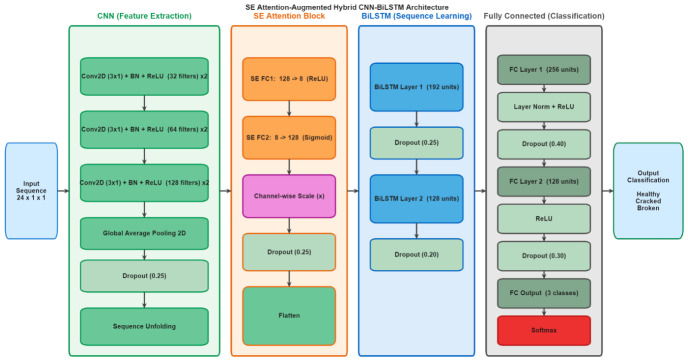
Proposed CNN-BiLSTM-SE hybrid architecture for detecting insulator defects.

Figure 7 shows the proposed hybrid CNN-BiLSTM architecture for detecting insulator break/crack conditions.

The proposed architecture accepts [24 × 1 × 1]-dimensional feature sequences as input in the first stage and uses a sequence folding layer to prepare the data for convolutional layers. The spatial feature extractor block consists of three main convolutional units organized hierarchically, with filter depths increasing gradually in the sequence 32, 64, and 128. Each unit contains consecutive Conv2D layers with [3 × 1] kernel sizes, while Batch Normalization is integrated to ensure training stability, and ReLU activation is incorporated to emphasize non-linearity. While the size reduction was made with the Global Average Pooling 2D layer located at the end of the block, the risk of overfitting of the model was minimized with a Dropout layer of 25%.

The SE block is applied to the output of global average pooling to recalibrate the 128-channel feature maps produced by the CNN on a channel-by-channel basis. During the squeeze phase, the global average value of each channel is calculated to obtain channel-wise statistics [28]:

(7) $$z_{c}=F_{sq}\left(u_{c}\right)=\frac{1}{H\times W}\sum_{i=1}^{H}\sum_{j=1}^{W}u_{c}(i,j)$$

During the excitation phase, a channel weight vector s in the range of 0–1 is generated for each channel via two complete linkage layers [28]:

(8) $$s=F_{ex}\left(z,W\right)=\sigma (W_{2}\delta \left(W_{1}z\right))$$

where z represents the squeeze output, *s* the weight scores assigned to each channel, *δ* the ReLU activation, *σ* the sigmoid activation, and *W*_1_ and *W*_2_ the weight matrices of the constriction and expansion full junction layers, respectively. In this study, the compression ratio was determined as r = 16; accordingly, the 128-channel CNN output was first reduced to 8 units, and then expanded back to 128 units. The generated s weight vector is multiplied element-by-element with CNN feature maps via the Channel-wise Scale layer, ensuring selective focusing on informative channel responses before proceeding to the BiLSTM stage.

The attributes reweighted by the SE mechanism are transferred to the temporal learning stage following the Sequence Unfolding and Flatten operations. At this stage, two consecutive BiLSTM layers are used. The first BiLSTM layer with 192 hidden units operates in sequence output mode, transferring information to the next layer, while the second BiLSTM layer with 128 hidden units produces a feature vector summarizing the final state of the entire sequence. Following each BiLSTM layer, dropout with rates of 25% and 20% is applied, respectively.

The classification block, which is the final stage of the architecture, consists of two fully connected layers (FC Layers) with 256 and 128 units, respectively, to perform feature fusion. In these layers, layer normalization and ReLU activation functions were chosen to optimize the network’s learning performance. The output layer of the model features a three-class softmax activation function representing the health status of porcelain insulators (healthy, broken, and cracked).

Table 5 provides a description of the network architecture used and the hyperparameters set for this model.

The dataset was partitioned into training (80%), validation (10%), and test (10%) subsets using stratified sampling to preserve class balance. To prevent any risk of data leakage arising from the overlapping windows, this partition was performed at the signal-block level prior to windowing: each continuous recording was divided along its time axis into contiguous training, validation, and test segments, and the overlapping windows were extracted independently within each segment. This guarantees that no overlapping windows are shared across the training, validation, and test sets. To mitigate the adverse effects of class imbalance on model learning, median-based class weighting was applied during training. All models were trained using the Adam optimization algorithm for a maximum of 250 epochs with a mini-batch size of 128. The initial learning rate was set to 3 × 10^−4^ and reduced by a factor of 0.5 every 50 epochs following a piecewise learning rate schedule. The hyperparameters listed in Table 5 were not obtained through an automated search; they were selected via a manual, validation-guided tuning procedure, starting from values commonly reported for CNN–BiLSTM architectures and refined to maximize validation accuracy while avoiding overfitting.

## 4. Results and Discussion

### 4.1. Statistical Validation and Feature Discriminability Analysis of the Leakage Current Dataset

The break and crack defects formed on a three-unit porcelain insulator string were tested under 60 kV voltage, and a data set was created. The empirical results of the Lilliefors (normality), Levene (homogeneity of variance), and Kruskal–Wallis (intergroup difference) tests, applied to verify the statistical validity of the leakage current dataset and the discrimination of the features, are presented in Table 6.

The Lilliefors test revealed a statistically significant violation of the assumption of normal distribution in all data sets (*p* < 0.001). Additionally, the Levene test results confirmed that the assumption of homogeneity of variance was also not met (*p* < 0.001). These findings support the use of the Kruskal–Wallis (KW) test, a non-parametric approach for analyzing intergroup differences, instead of parametric tests. These violations are physically expected: discharge activity in cracked and broken insulators is intermittent and impulsive, as surface defects create localized high-field regions where partial discharges and micro-arcs occur sporadically, generating sudden current bursts of irregular magnitude. This produces skewed, heavy-tailed feature distributions rather than Gaussian ones, while the discharge severity—and hence the feature variance—differs strongly between the healthy, cracked, and broken conditions. The distribution-free Kruskal–Wallis test is therefore the statistically appropriate choice for comparing the three conditions.

According to the Kruskal–Wallis test results, the cracked group obtained a value of 0.6398 for the amplitude attribute. This statistical finding indicates that despite the formation of a crack in the insulator, there was no significant deviation in the leakage current amplitude. It is understood that the surface cracks have not yet fully shortened the current path and have not sufficiently disrupted dielectric integrity to radically alter the magnitude of the leakage current. Analyses conducted on the 3rd and 5th harmonic components of the leakage current signal produced statistically highly significant results (*p* < 0.001) across all fault scenarios. This finding constitutes the rationale for selecting harmonic components representing the nonlinear discharge characteristic on the insulator surface as the key discriminatory features of the fault detection algorithm.

**Figure 8:**
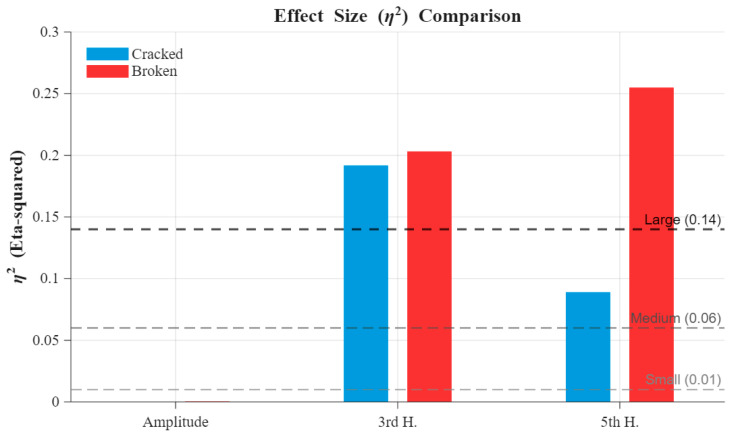
Comparative analysis of leakage current characteristics in terms of effect size for different insulator defect conditions.

In Figure 8, a comparison of effect sizes is made.

The 3rd harmonic component is the most consistent discriminator, exceeding the large-effect threshold (η^2^ > 0.14) in both the cracked (η^2^ = 0.1918) and broken (η^2^ = 0.2032) scenarios. The behaviour of the 5th harmonic is defect-dependent: it shows a medium effect for cracks (η^2^ = 0.0890) but a large effect for breaks (η^2^ = 0.2550), where surface fragmentation intensifies the nonlinear discharge behaviour and amplifies higher-order harmonic distortion. By contrast, the raw amplitude exhibits a negligible effect size in both cases (η^2^ ≤ 0.0005), confirming that the discriminative information resides in the harmonic structure rather than in the signal magnitude. The analysis was deliberately restricted to harmonic orders below the Nyquist frequency (333.3 Hz) to ensure spectral validity.

**Figure 9:**
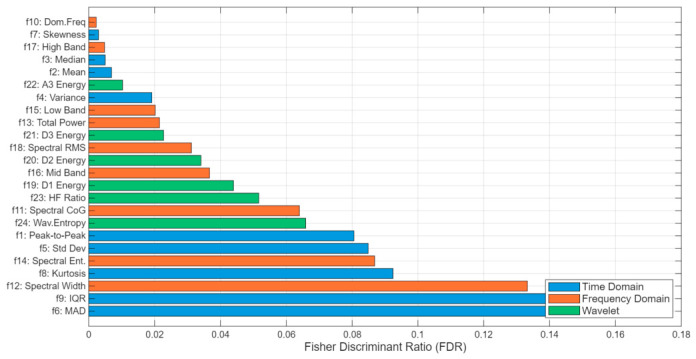
Fisher Discriminant Ratio (FDR) analysis of all 24 extracted features grouped by domain.

The discriminatory powers of the 24 extracted features among insulator fault conditions were evaluated using Fisher Discriminant Ratio (FDR) analysis, and the results are presented in Figure 9 [55,56].

As shown in Figure 9, among time-domain features, f6 (MAD), f9 (IQR) and f8 (Kurtosis) exhibit the highest discriminability, reflecting the amplitude irregularities introduced by surface discharge events. In the frequency domain, f12 (Spectral Width) and f14 (Spectral Entropy) demonstrate strong discriminative power, consistent with the Kruskal–Wallis findings confirming the significance of harmonic components. Among wavelet features, f23 (HF Ratio) and f24 (Wavelet Entropy) show notable discriminability, further validating the complementary role of wavelet decomposition in capturing transient discharge characteristics. Thus, the FDR analysis provides a discriminability ranking across all 24 features, while the subsequent Kruskal–Wallis test offers a formal significance assessment of the most informative harmonic components, together forming a comprehensive statistical justification for the selected feature set.

**Figure 10:**
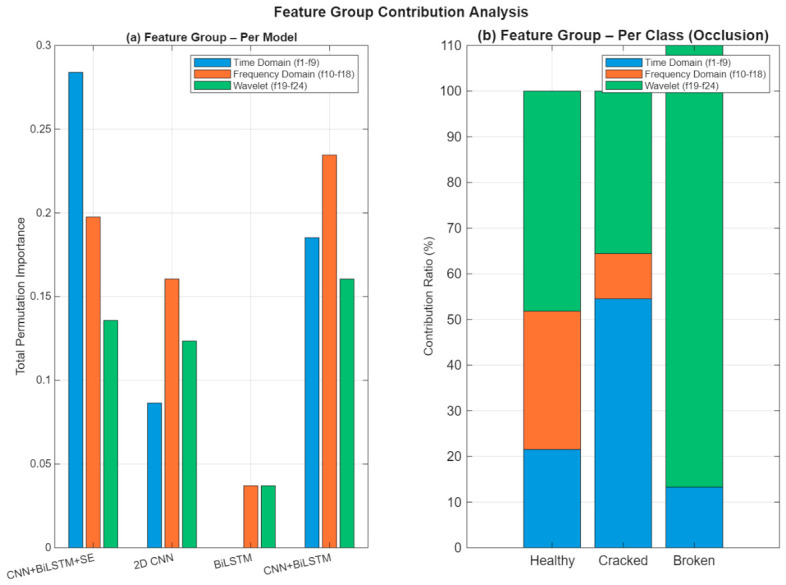
Feature group contribution analysis: (**a**) model-specific permutation importance across the four architectures, and (**b**) class-wise occlusion-based attribution for the proposed CNN–BiLSTM–SE model.

To examine the contribution of each feature group to the model’s decisions, two complementary attribution analyses were performed: a permutation importance analysis across all four model architectures and an occlusion-based attribution analysis for the proposed CNN + BiLSTM + SE model. The results are presented in Figure 10. For the permutation importance analysis, each feature was randomly shuffled across the test instances and passed through the trained model, with the resulting accuracy drop taken as its importance; the procedure was applied to the test set using the fixed trained model.

As shown in Figure 10a, the CNN + BiLSTM + SE model exhibits the highest total permutation importance across all three feature groups—time domain (0.284), frequency domain (0.199), and wavelet (0.135)—demonstrating its superior capacity to leverage complementary information from all signal domains. To interpret the model’s decisions at the class level, an occlusion-based attribution analysis was performed (Figure 10b), in which each input feature is systematically masked and the resulting change in the class score is measured. This analysis shows that the model relies on different feature groups for different conditions: the cracked condition is driven predominantly by time-domain features (53.6%), the broken condition is dominated by wavelet-domain features (88.2%), and the healthy condition draws on a more balanced combination of wavelet (45.2%) and frequency-domain (31.5%) features. The consistency between the two independent attribution methods confirms that the model’s decisions are grounded in physically meaningful signal characteristics rather than spurious correlations.

### 4.2. Classification Results and Performance Comparison of Deep Learning Architectures

**Figure 11:**
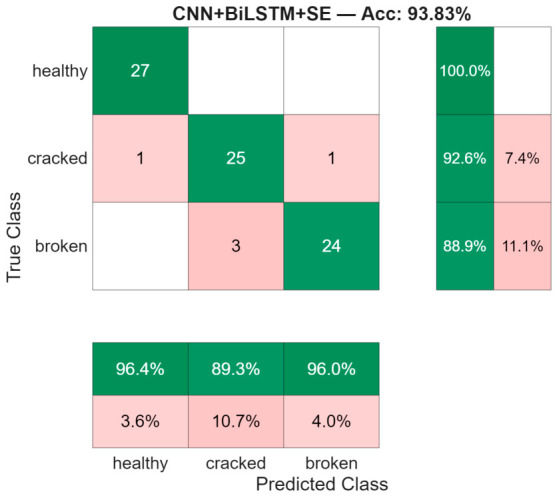
Confusion matrix of the proposed hybrid CNN–BiLSTM–SE model.

Experimental studies were conducted on healthy, cracked, and fractured porcelain insulator units under 60 kV voltage to generate a data set for fault diagnosis. The inter-class discrimination performance of the developed CNN-BiLSTM architecture on the complex dataset is presented in the confusion matrix in Figure 11.

When examining Figure 11, the healthy insulator class achieved a 100.0% recall rate with all 27 samples correctly classified. In the cracked class, 25 out of 27 samples were correctly classified, with 1 sample misassigned to the healthy class and 1 to the broken class (92.6% recall). In the broken class, 24 out of 27 samples were correctly classified, with 3 samples confused with the cracked class (88.9% recall).

**Figure 12:**
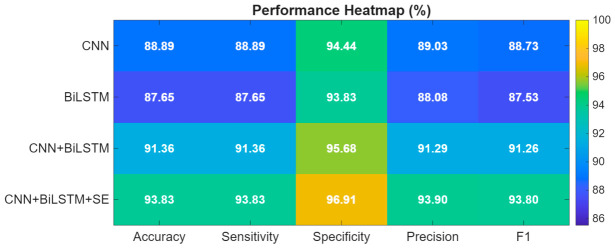
Comparative analysis of success metrics in porcelain insulator defect diagnosis of different deep learning architectures.

The performance heat map presented in Figure 12 compares the accuracy, sensitivity, specificity, precision, and F1-score metrics of the four models in a comparative format.

The CNN + BiLSTM + SE model achieved the highest performance across all metrics, attaining 93.83% accuracy, 93.83% sensitivity, 96.91% specificity, 93.90% precision, and 93.80% F1-score. The specificity value of 96.91% in particular demonstrates the model’s ability to reliably identify healthy insulators. The standalone CNN model achieved 88.89%, the BiLSTM model 87.65%, and the CNN + BiLSTM model 91.36% accuracy. The fact that the CNN + BiLSTM + SE model outperforms the CNN + BiLSTM model by 2.47%, the standalone CNN model by 4.94%, and the BiLSTM model by 6.18% numerically confirms the significant contribution of the SE attention mechanism to classification performance.

To further benchmark the proposed model against conventional approaches, three classical machine-learning algorithms—an SVM with an RBF kernel, a Random Forest, and a boosted-tree ensemble—were trained on the same 24-feature representation and evaluated on the same test set. Their accuracies are summarized in Table 7 [41,44].

As shown in Table 7, all three classical baselines remain below the proposed model. The best-performing classical method, Random Forest (90.12%), reaches a level comparable to the standalone deep models given in Figure 12 (CNN: 88.89%; CNN–BiLSTM: 91.36%), confirming that the engineered 24-feature representation is itself informative. The additional improvement of the proposed CNN–BiLSTM–SE demonstrates that the SE attention mechanism integrated into the hybrid architecture provides discriminative power that neither the classical models nor the plain deep models capture from the same features.

**Figure 13:**
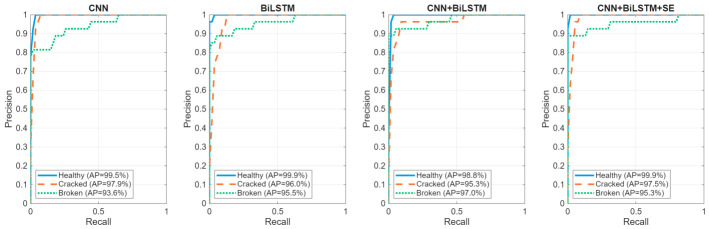
Comparison of Precision–Recall (PR) curves and average precision (AP) values for the porcelain insulator defect classes of the CNN, BiLSTM, CNN + BiLSTM, and proposed hybrid CNN–BiLSTM–SE models.

The Precision–Recall (PR) curves and Average Precision (AP) values presented in Figure 13 provide a comparative analysis of the proposed CNN–BiLSTM–SE method with independent model architectures.

When examining the PR curves, it is observed that the CNN + BiLSTM + SE model reaches the highest value with 99.9% AP for the healthy class. In the crack-containing class, the CNN + BiLSTM + SE model achieved 97.5% AP, while the CNN model slightly outperformed it with 97.9% AP; this reflects the decisive role of frequency domain features in crack detection and the subtle performance difference between the models. Among the fracture class models, the CNN + BiLSTM model achieved the highest value with 97.0% AP, while the CNN + BiLSTM + SE model exhibited a similar performance with 95.3% AP. Overall, all models demonstrated high AP values; the proposed CNN + BiLSTM + SE architecture, on the other hand, offers balanced and stable performance across all classes.

To further verify the robustness of the proposed model and to ensure that the reported performance is not dependent on a single data partition, a 5-fold stratified cross-validation was carried out. The mean and standard deviation of the performance metrics across the five folds are summarized in Table 8.

As shown in Table 8, the model maintains a consistently high accuracy across all folds, with a standard deviation of only 2.02%. The per-fold accuracies (87.12%, 90.24%, 90.24%, 92.07%, and 87.80%) confirm that the performance is stable, and the single-split result of 93.83% reported above falls within the upper range of these folds, demonstrating that it is a representative estimate.

The computational efficiency of the proposed model was also evaluated, as real-time monitoring is a key objective of this work. The CNN–BiLSTM–SE architecture comprises approximately 1.18 million trainable parameters. On the workstation described in Section 2.3, it achieves an average inference time of 12.78 ms per signal segment in single-sample mode and 0.77 ms per segment in batch mode, corresponding to a throughput of about 1300 segments per second. As each segment represents roughly 7.5 s of acquisition, the inference latency is negligible relative to the acquisition time, confirming the suitability of the model for real-time, online insulator condition monitoring.

### 4.3. Discussion and Limitations

The proposed approach can be positioned within three main streams of insulator condition monitoring. The first and most prevalent stream relies on image-based detection using aerial or thermographic imagery with object-detection networks such as YOLO and R-CNN variants [1,16,17,24]. While effective for detecting visible mechanical damage, these methods require optical access to the insulator and cannot capture the electrical degradation processes that precede visible failure. The second stream uses leakage-current analysis with classical or shallow learning, such as ANN-based classification of ceramic insulators [17], supervised condition monitoring [41], and waveform classification [46]; these approaches confirm the diagnostic value of leakage current but typically rely on hand-crafted decision rules or single-stage classifiers. The third and most closely related stream applies hybrid deep architectures to leakage current, notably CNN–LSTM and CNN–BiLSTM models for online insulator classification [16,53] and CNN–BiLSTM for infrared image classification [38]. Our work extends this third stream in two respects: (i) it integrates a Squeeze-and-Excitation channel-attention mechanism [29] into the CNN–BiLSTM backbone, allowing the network to adaptively recalibrate the relative importance of the extracted feature channels—a component absent from the CNN–LSTM/BiLSTM models in [16,40,53]; and (ii) it couples the classifier with a statistical-explainability framework (FDR, Kruskal–Wallis, permutation and occlusion attribution), whereas most prior leakage-current studies report classification performance without interpreting the physical basis of the decisions. A direct numerical accuracy comparison with these studies is deliberately avoided, since they employ different datasets and no shared benchmark dataset is yet available for this problem; the comparison is therefore framed at the methodological level.

Despite the promising results, one principal limitation should be acknowledged. All data were obtained under controlled laboratory conditions from a single experimental setup. Real-world insulators are additionally subject to environmental stressors—such as pollution, humidity, salt deposition, and temperature cycling—that are known to alter leakage-current behaviour [13,45]. Consequently, the performance of the proposed model under such unseen field conditions remains to be validated. The acquisition of multi-site field data and the evaluation of the model under real operating conditions therefore constitute the primary direction for future work. Although the proposed methodology is transferable to other insulator types given suitable training data, the specific crack- and break-monitoring scheme studied here is most meaningful for porcelain insulators: glass insulators typically shatter completely rather than developing observable cracks, and silicone-rubber composite insulators do not undergo such brittle cracking or breakage, degrading instead through different mechanisms.

## 5. Conclusions

This study proposes an explainable hybrid deep learning architecture based on leakage current signals, focusing on the structural damage detection of porcelain insulators that exhibit high sensitivity to sudden temperature fluctuations caused by global climate change. Experimental studies and analyses conducted to diagnose healthy, cracked, and broken structural conditions in three-unit porcelain insulators have revealed the following key findings:

A comprehensive dataset consisting of 15 different scenarios covering all possible combinations of healthy, cracked, and broken states based on the structural integrity of insulator units has been designed. The raw leakage current signals obtained under 60 kV voltage were pre-processed with the Savitzky–Golay filter to suppress noise while preserving waveform continuity.

To verify the statistical validity of the leakage current dataset, the Lilliefors, Levene, and Kruskal–Wallis tests were applied. The 3rd and 5th harmonic components were statistically proven to play a critical role in insulator damage detection: the 3rd harmonic exceeded the large-effect threshold (η^2^ > 0.14) in both defect types, while the 5th harmonic reached a large effect in the break case (η^2^ = 0.2550).

The proposed CNN–BiLSTM–SE architecture enhances CNN-based spatial feature extraction with BiLSTM-based temporal dependency modeling through an SE channel attention mechanism. The SE block enhances classification performance by focusing on the most discriminative channel responses before proceeding to the BiLSTM stage. It was found that adding the SE attention block provided a 2.47% accuracy increase compared to the CNN + BiLSTM baseline. The proposed CNN–BiLSTM–SE architecture achieved a 93.83% accuracy rate in general classification, outperforming the CNN model by 4.94% and the BiLSTM model by 6.18%. Furthermore, the proposed model outperformed classical machine-learning baselines (SVM: 87.65%, Random Forest: 90.12%, Boosted Trees: 87.65%), confirming that the performance gain stems from the SE-augmented hybrid architecture rather than from the engineered features alone.

The reliability and interpretability of the proposed model were further established. Five-fold stratified cross-validation confirmed the stability of the results, yielding a mean accuracy of 89.50% ± 2.02% across folds. The model’s decisions were interpreted through Fisher Discriminant Ratio analysis together with permutation- and occlusion-based attribution, which showed that the network relies on physically meaningful feature groups rather than spurious correlations. With approximately 1.18 million parameters and an inference time of 0.77 ms per segment, the model is also suitable for real-time, online insulator monitoring.

As the present study was conducted under controlled laboratory conditions using a single experimental setup, validation of the model under unseen field conditions—involving environmental factors such as pollution, humidity, and temperature variation—constitutes the primary direction for future work.

## Figures and Tables

**Table 1:** Experimental situations and insulator unit configurations.

Situation Number	Unit 1 (Top)	Unit 2 (Middle)	Unit 3 (Bottom)
1	Healthy	Healthy	Healthy
2	Cracked	Healthy	Healthy
3	Healthy	Cracked	Healthy
4	Healthy	Healthy	Cracked
5	Cracked	Cracked	Healthy
6	Cracked	Healthy	Cracked
7	Healthy	Cracked	Cracked
8	Cracked	Cracked	Cracked
9	Broken	Healthy	Healthy
10	Healthy	Broken	Healthy
11	Healthy	Healthy	Broken
12	Broken	Broken	Healthy
13	Broken	Healthy	Broken
14	Healthy	Broken	Broken
15	Broken	Broken	Broken

**Table 2:** Time-Domain Features.

Feature	Equation	Description and Characteristic	Its Role in Defect Detection
*f*_1_	$f_{1}=\max\left(x_{i}\right)-min(x_{i})$	It is the difference between the maximum and minimum values of the signal.	Identifies abrupt amplitude variations.
*f*_2_	$f_{2}=\frac{1}{N}\sum_{i=1}^{N}x_{i}$	It is the arithmetic mean of the signal.	It monitors average current changes.
*f*_3_	$f_{3}=\mathrm{median}\left(X\right)$	It is the value at the very center of the data set.	It maintains the base level without being affected by sudden changes.
*f*_4_	$f_{4}=\frac{1}{N-1}\sum_{i=1}^{N}{\left(x_{i}-x\right)}^{2}$	It is the average spread square of the values.	Measures energy fluctuations caused by leakage current.
*f*_5_	$f_{5}=\sqrt{f_{4}}$	Is the square root of the variance.	Determines the overall level of irregularity in the signal.
*f*_6_	$f_{6}=median(\left|x_{i}-\mathrm{median}\left(X\right)\right|)$	It is the median of their absolute distance from the median.	It is effective against noise in the detection of arc welding-related leakage currents.
*f*_7_	$f_{7}=\frac{1}{N}\sum_{i=1}^{N}\frac{{\left(x_{i}-x\right)}^{3}}{(N-1)\sigma^{3}}$	It shows how much the distribution deviates from symmetry.	It detects asymmetric disturbances in leakage current paths.
*f*_8_	$f_{8}=\frac{1}{N}\sum_{i=1}^{N}\frac{{\left(x_{i}-x\right)}^{4}}{(N-1)\sigma^{4}}$	Measures the steepness of the distribution and the influence of extreme values.	Detects sudden arc types.
*f*_9_	$f_{9}=Q_{3}-Q_{1}$	It is the difference between the third and first quarters.	It is used to analyze the true signal propagation in leakage current signals by eliminating background noise.

**Table 3:** Frequency-Domain Features.

Feature	Equation	Description and Characteristic	Its Role in Defect Detection
*f*_10_	$f_{10}=argmaxP(f)$	It is the frequency with the highest amplitude in the power spectrum.	It monitors shifts in resonance points.
*f*_11_	$f_{11}=\frac{\sum_{i=1}^{M}f_{i}P{(f}_{i})}{\sum_{i=1}^{M}P{(f}_{i})}$	It is the center of gravity of the signal on the frequency axis.	It monitors the shift in energy to high-frequency harmonics during a fault.
*f*_12_	$f_{12}=\sqrt{\frac{\sum_{i=1}^{M}{\left(f_{i}-f_{11}\right)}^{2}P{(f}_{i})}{\sum_{i=1}^{M}P{(f}_{i})}}$	It is the distribution of frequency components around the average frequency.	It shows how wide a frequency band the signal is spread over.
*f*_13_	$f_{13}=\sum_{i=1}^{M}P{(f}_{i})$	It is the total power of all frequencies within the spectrum.	It measures the total electrical load added to the system by the leakage current.
*f*_14_	$f_{14}=-\sum_{i=1}^{M}p_{i}lnp_{i}$	It is the degree of irregularity of the spectral energy distribution.	It determines the complexity of chaotic and noisy events such as arc faults.
*f*_15_	$f_{15}=\sum P(f)$, f < 66.7 Hz	It is the total power of components below 0.1 Hz.	It captures very slow system oscillations.
*f*_16_	$f_{16}=\sum P(f)$, 66.7 ≤ f ≤ 200 Hz	It is the total power of the components between 0.1 Hz and 0.3 Hz.	Monitors system interactions.
*f*_17_	$f_{17}=\sum P(f)$, f > 200 Hz	It is the power of high-frequency components above 0.3 Hz.	It detects high-frequency arcs and harmonics.
*f*_18_	$f_{18}=\sqrt{\frac{1}{M}\sum_{i=1}^{M}{\left[P(f_{i})\right]}^{2}}$	It is the root mean square of the power spectral density values.	Measures general spectral intensity and broadband noise.

**Table 4:** Wavelet-Domain Features.

Feature	Equation	Description and Characteristic	Its Role in Defect Detection
*f*_19_	$f_{19}=\sum_{k=1}^{N_{1}}{\left(d_{1},k\right)}^{2}$	The energy of the 1st level detail coefficients obtained by wavelet decomposition.	It captures high-frequency transient components associated with discharge events on the insulator surface.
*f*_20_	$f_{20}=\sum_{k=1}^{N_{2}}{\left(d_{2},k\right)}^{2}$	The energy of the 2st level detail coefficients obtained by wavelet decomposition.	It detects medium-frequency oscillations resulting from partial discharge activity and microcrack formation.
*f*_21_	$f_{21}=\sum_{k=1}^{N_{3}}{\left(d_{3},k\right)}^{2}$	The energy of the 3st level detail coefficients obtained by wavelet decomposition.	It detects low-frequency structural changes resulting from the progressive degradation of the insulator.
*f*_22_	$f_{22}=\sum_{k=1}^{N_{a}}{\left(a_{3},k\right)}^{2}$	The energy of the 3rd level approximation coefficients obtained by wavelet decomposition.	It represents the fundamental frequency component of the ground fault current signal and the overall energy level.
*f*_23_	$f_{23}=\frac{f_{19}+f_{20}}{f_{19}+f_{20}{+f}_{21}+f_{22}}$	The ratio of high-frequency detail energy to the total wavelet energy across all decomposition levels.	It measures the dominance of high-frequency discharge components; it increases significantly under faulty insulator conditions.
*f*_24_	$f_{24}=-\sum_{j=1}^{3}p_{j}\log_{2}\left(p_{j}\right)$	Wavelet entropy based on normalized subband energy distribution.	It measures signal complexity across separation levels; healthy insulators exhibit low entropy values, while broken insulators show significantly higher entropy values.

**Table 5:** Description of the proposed CNN-BiLSTM-SE architecture.

Layer Type	Maps/Units	Kernel/Stride	Output Size	Activation
Input	-	-	24 × 1 × 1	-
Conv2D (1a, 1b)	32	3 × 1/1	24 × 1 × 32	ReLU
Batch Normalization	-	-	24 × 1× 32	-
Conv2D (2a, 2b)	64	3 × 1/1	24 × 1× 64	ReLU
Batch Normalization	-	-	24 × 1 × 64	-
Conv2D (3a, 3b)	128	3 × 1/1	24 × 1 × 128	ReLU
Batch Normalization			24 × 1× 128	
Global Avg Pool	-	-	1 × 1 × 128	-
SE FC1 (Squeeze)	8	-	8	ReLU
SE FC2 (Excitation)	128	-	128	Sigmoid
Channel—wise Scale	-	-	1 × 1 × 128	-
Dropout (CNN)	-	-	128	-
Flatten	-	-	128	-
BiLSTM 1	192	-	50 × 384	-
BiLSTM 2	128	-	256	-
Fully Connected 1	256	-	256	ReLU
Layer Normalization	-	-	256	-
Fully Connected 2	128	-	128	ReLU
Fully Connected (Out)	3	-	3	Softmax

**Table 6:** Statistical validation results for leakage current features.

Statistical Test	Condition	*p*-Value
Lilliefors	Cracked	*p* < 0.001
	Broken	*p* < 0.001
Levene	Cracked	*p* < 0.001
	Broken	*p* < 0.001
Kruskal–Wallis (Amplitude)	Cracked	*p* = 0.6398
	Broken	*p* < 0.001
Kruskal–Wallis (3rd Harmonic)	Cracked	*p* < 0.001
	Broken	*p* < 0.001
Kruskal–Wallis (5th Harmonic)	Cracked	*p* < 0.001
	Broken	*p* < 0.001

**Table 7:** Performance comparison between classical machine-learning baselines and the proposed model on the test set.

Model	Accuracy (%)
SVM	87.65
Boosted Trees	87.65
Random Forest	90.12
**Proposed**	**93.83**

**Table 8:** Five-fold cross-validation results of the proposed CNN–BiLSTM–SE model.

Metric	Mean (%)	Std (%)
Accuracy	89.50	2.02
Sensitivity	89.51	2.01
Specificity	94.75	1.01
Precision	89.71	1.92
F1-score	89.48	2.00

## Data Availability

The data presented in this study is available on request from the corresponding author.
